# Supplementary material for: TANGO: Analysis and curation of particles in cryo-electron tomography
Source: Nat Commun. 2026 Feb 10;17:1557. doi: 10.1038/s41467-026-69195-5 (PMC12894703; doi:10.1038/s41467-026-69195-5)
Supplement: Supplementary file 2 — Reporting Summary [file 41467_2026_69195_MOESM2_ESM.pdf]

nature portfolio

Corresponding author(s): Beata Turonova

Last updated by author(s): 06.12.2025

Reporting Summary

Nature Portfolio wishes to improve the reproducibility of the work that we publish. This form provides structure for consistency and transparency in reporting. For further information on Nature Portfolio policies, see our Editorial Policies and the Editorial Policy Checklist.

Statistics

For all statistical analyses, confirm that the following items are present in the figure legend, table legend, main text, or Methods section.

n/a	Confirmed	
☐	☒	The exact sample size (*n*) for each experimental group/condition, given as a discrete number and unit of measurement
☒	☐	A statement on whether measurements were taken from distinct samples or whether the same sample was measured repeatedly
☐	☒	The statistical test(s) used AND whether they are one- or two-sided<br>*Only common tests should be described solely by name; describe more complex techniques in the Methods section.*
☒	☐	A description of all covariates tested
☒	☐	A description of any assumptions or corrections, such as tests of normality and adjustment for multiple comparisons
☐	☒	A full description of the statistical parameters including central tendency (e.g. means) or other basic estimates (e.g. regression coefficient) AND variation (e.g. standard deviation) or associated estimates of uncertainty (e.g. confidence intervals)
☐	☒	For null hypothesis testing, the test statistic (e.g. *F*, *t*, *r*) with confidence intervals, effect sizes, degrees of freedom and *P* value noted<br>*Give P values as exact values whenever suitable.*
☒	☐	For Bayesian analysis, information on the choice of priors and Markov chain Monte Carlo settings
☒	☐	For hierarchical and complex designs, identification of the appropriate level for tests and full reporting of outcomes
☒	☐	Estimates of effect sizes (e.g. Cohen's *d*, Pearson's *r*), indicating how they were calculated

Our web collection on statistics for biologists contains articles on many of the points above.

Software and code

Policy information about availability of computer code

Data collection	No data collection was done specifically for this study.
Data analysis	IMOD (v4.9), ChimeraX (v1.5), ArtiaX (v0.4.2), novaSTA (v1.1) , cryoCAT (v0.6.1), cryoCAT (v0.7.1), the code developed within this manuscript is available on github as part of the cryoCAT repository: https://github.com/turonova/cryocat

For manuscripts utilizing custom algorithms or software that are central to the research but not yet described in published literature, software must be made available to editors and reviewers. We strongly encourage code deposition in a community repository (e.g. GitHub). See the Nature Portfolio guidelines for submitting code & software for further information.

Data

Policy information about availability of data

All manuscripts must include a data availability statement. This statement should provide the following information, where applicable:

- Accession codes, unique identifiers, or web links for publicly available datasets
- A description of any restrictions on data availability
- For clinical datasets or third party data, please ensure that the statement adheres to our policy

No primary data was produced for this study. All STA maps from the supplementary information are available on Zenodo [https://doi.org/10.5281/zenodo.17843887]. The EMDB entries that were used for the visualization in this study are EMD-51626 [http://doi.org/doi:10.1016/j.cell.2024.12.008] (in situ human cytoplasmic ring subunit of nuclear pore complex), EMD-63079 [http://doi.org/doi:10.1111/gtc.70019] (native HeLa nucleosome in poly-nucleosomes (class 1)), EMD-13365 [http://doi.org/doi:10.1038/s41594-022-00768-w] (nucleosome stack of the 4x187 nucleosome array containing H1), EMD-13363 [http://doi.org/

doi:10.1038/s41594-022-00768-w] (trinucleosome of the 4x177 nucleosome array containing H1), EMD-2601 [http://doi.org/doi:10.1126/science.1251413] (double helix twisted by tetranucleosomes), EMD-16721 [http://doi.org/doi:10.1126/science.adh1411] (human 80S ribosome), EMD-10398 [http://doi.org/doi:10.15252/embj.2019103365] (yeast di-ribosome stalled on poly(A) tract). Furthermore, tomograms reconstructed from tilt series with accession code EMPIAR-10277 [https://doi.org/10.6019/EMPIAR-10277] were used for STA of HIV-1 CA-SP1 VLPs, and a tomogram reconstructed from tilt series with accession code EMPIAR-11538 [https://doi.org/10.6019/EMPIAR-11538] was used for STA of microtubules. The source data underlying Figures 2d, e, 3f, 4c, d, e, f, 5c, d, e, g, h, 6, Supplementary Figure 7, and Supplementary Tables 3 and 4 are provided as a Source Data file.

Research involving human participants, their data, or biological material

Policy information about studies with human participants or human data. See also policy information about sex, gender (identity/presentation), and sexual orientation and race, ethnicity and racism.

Reporting on sex and gender	Not applicable.
Reporting on race, ethnicity, or other socially relevant groupings	Not applicable.
Population characteristics	Not applicable.
Recruitment	Not applicable.
Ethics oversight	Not applicable.

Note that full information on the approval of the study protocol must also be provided in the manuscript.

Field-specific reporting

Please select the one below that is the best fit for your research. If you are not sure, read the appropriate sections before making your selection.

[x] Life sciences [ ] Behavioural & social sciences [ ] Ecological, evolutionary & environmental sciences

For a reference copy of the document with all sections, see nature.com/documents/nr-reporting-summary-flat.pdf

Life sciences study design

All studies must disclose on these points even when the disclosure is negative.

Sample size	Not applicable.
Data exclusions	Not applicable.
Replication	Not applicable.
Randomization	Not applicable.
Blinding	Not applicable.

Reporting for specific materials, systems and methods

We require information from authors about some types of materials, experimental systems and methods used in many studies. Here, indicate whether each material, system or method listed is relevant to your study. If you are not sure if a list item applies to your research, read the appropriate section before selecting a response.

Materials & experimental systems

n/a	Involved in the study	
[x]	[ ]	Antibodies
[x]	[ ]	Eukaryotic cell lines
[x]	[ ]	Palaeontology and archaeology
[x]	[ ]	Animals and other organisms
[x]	[ ]	Clinical data
[x]	[ ]	Dual use research of concern
[x]	[ ]	Plants

Methods

n/a	Involved in the study	
[x]	[ ]	ChIP-seq
[x]	[ ]	Flow cytometry
[x]	[ ]	MRI-based neuroimaging

Plants

Seed stocks	Not applicable.
Novel plant genotypes	Not applicable.
Authentication	Not applicable.
